# Association between Vascular Calcification and Esophagojejunal Anastomotic Complications after Total Gastrectomy for Gastric Cancer: A Propensity-Matched Study

**DOI:** 10.3390/curroncol29050262

**Published:** 2022-05-03

**Authors:** Su-Lim Lee, Chul-Hyo Jeon, Ki-Bum Park, Ho-Seok Seo, Han-Hong Lee

**Affiliations:** 1 Department of Radiology, Uijeongbu St. Mary’s Hospital, College of Medicine, The Catholic University of Korea, 271, Cheonbo-Ro, Uijeongbu-si 11765, Korea; radlsl@catholic.ac.kr; 2 Division of Gastrointestinal Surgery, Department of Surgery, Seoul St. Mary’s Hospital, College of Medicine, The Catholic University of Korea, 222, Banpo-daero, Seocho-gu, Seoul 06591, Korea; bfofsyr@naver.com (C.-H.J.); apzzap2@gmail.com (K.-B.P.); comaguitar@catholic.ac.kr (H.-S.S.)

**Keywords:** gastrectomy, morbidity, stomach neoplasm, vascular calcification

## Abstract

Esophagojejunal anastomosis (EJA) complications after total gastrectomy are related to significant morbidity and mortality. The aim of this study was to evaluate the association between arterial calcifications and EJA complications such as leak and stricture for gastric cancer. Between January 2014 and October 2019, 30 patients with EJA complications after total gastrectomy were enrolled and matched to 30 patients without complications through retrospective data review. Arterial calcification grade on preoperative computed tomography (CT) was reported in the abdominal aorta and superior mesenteric artery (SMA) as “absent”, “minor”, or “major”, and in the jejunal vascular arcade (JVA) and left inferior phrenic artery (LIPA) as “absent” or “present”. A Chi-square test was used to compare the variables between the two groups. *p*-Value < 0.050 was considered statistically significant. Among 30 patients, the numbers of patients with leak and stricture were 23 and seven, respectively. Aortic calcifications were not associated with EJA complications regardless of their grade (*p* = 0.440). Only major SMA calcifications were associated with EJA complications, as they were present in five patients (16.7%) in the complication group and absent in the non-complication group (*p* = 0.020). Major SMA calcifications were more related to anastomotic stricture than leak. Three (13.0%) out of 23 patients with leak and two (28.6%) out of seven with stricture had major SMA calcifications (*p* = 0.028). No calcifications were detected in the JVA or LIPA in any of the 60 patients. Major SMA calcifications were found to be associated with EJA complications, especially in stricture.

## 1. Introduction

Roux-en-Y esophagojejunal anastomosis (EJA) is commonly used for reconstruction after total gastrectomy for gastric cancer. Adequate blood supply is crucial for anastomotic site healing. After esophagojejunostomy, the jejunal side of the anastomosis is supplied by the jejunal vascular arcade (JVA), a branch of the superior mesenteric artery (SMA). As the left gastric and short gastric arteries are divided during total gastrectomy, the distal abdominal esophageal end is dependent only on intramural, submucosal blood flow in addition to the extramural blood supply via the left inferior phrenic artery (LIPA) which has variable sites of origin, such as the celiac trunk and aorta [1].

The anastomotic complications of esophagojejunostomy are associated with significant morbidity and mortality, increased length of hospital stay, and costs of medical care. Anastomotic leak is a life-threatening complication in the early postoperative period and is an independent negative predictive factor for long term survival in gastric cancer [2,3,4]. Anastomotic stricture can lead to dysphagia with an unfavorable effect on quality of life. In a meta-analysis which included 2484 patients who underwent total gastrectomy for cancer, anastomotic leak and stricture were reported in 2.5% and 2.9% of patients, respectively [5].

Several factors have been identified to increase the risk of anastomotic complications. Male sex, the presence of cardiovascular disease (CVD), perioperative transfusion, and tumor location were associated with anastomotic leak [6]. The cause of stricture is not well understood, but tension on the anastomosis, edema, and foreign-body reaction are recognized factors [7].

In this study, we attempted to find if an association exists between vascular calcifications and the incidence of EJA complications after total gastrectomy for gastric cancer using preoperative computed tomography (CT) assessment of the location and extent of calcifications.

## 2. Materials and Methods

### 2.1. Definitions of Complications

Anastomotic leak: diagnosed by the presence of clinical signs of tachycardia, fever, decreased urine output, abdominal pain, peritonitis, pus, or intestinal content from the abdominal drain, with radiologic evidence of contrast extravasation or endoscopic confirmation of anastomotic disruption.

Anastomotic stricture: diagnosed by narrowing with difficult or impossible passage of a gastroduodenoscope in symptomatic patients with dysphagia. All patients with stricture have no previous history of leak prior to their diagnosis with stricture.

### 2.2. Patients & Matching

Thirty patients who underwent total gastrectomy for gastric cancer in Seoul St. Mary’s hospital between January 2014 and October 2019 were enrolled and matched to 30 patients based on the occurrence of postoperative EJA leak or stricture. All operations were performed by specialized gastric cancer surgeons following Korean Gastric Cancer Association Guidelines [8]. The study was approved by the Institutional Review Board (IRB) of Seoul St. Mary’s hospital (approval No. KC20RASI0448), and the informed consent was waived due to the retrospective nature of the study.

A propensity score matching (PSM) was used at a 1:1 ratio to overcome the demographic and clinical differences between the two groups. The patients were matched regarding age, sex, Body Mass Index (BMI), history of CVD or smoking, diabetes mellitus (DM), surgical approach (open or laparoscopic), remnant gastrectomy, tumor location, and the pathological stage of disease. The BMI was categorized as “underweight” (<18.50), “normal” (18.50–22.99), “overweight” (23.00–29.99), and “obese” (≥30.00).

### 2.3. Anastomosis Technique

After laparoscopic total gastrectomy, the EJA was constructed by the overlap method using a 45 mm linear stapler between the left lower end of the esophagus and the jejunal loop, and the common entry hole was closed with a continuous barbed suture. In open technique we used a 25 mm circular stapler for EJA with interrupted absorbable suture reinforcement on the staple line. In both groups the Roux limb is 40 cm long. 

### 2.4. Image Acquisition and Evaluation 

**Figure 1:**
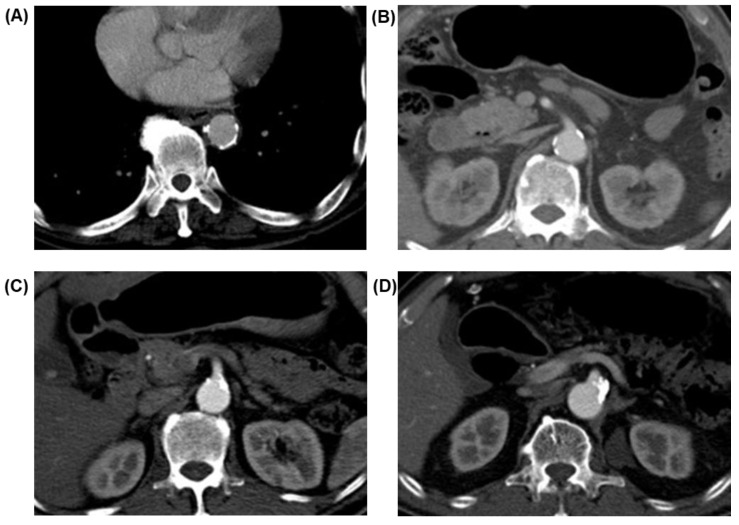
Examples of image characteristics of different grades of aortic and SMA calcifications (**A**) aorta/score 2, (**B**) superior mesenteric artery (SMA)/score 1, (**C**) SMA/score 2 and (**D**) SMA/score 2.

Image acquisition was performed using a 128-multidetector CT scanner. One pack of effervescent granules with a small amount of water was administered orally to distend the gastric lumen. CT was obtained after intravenous injection of 100–150 milliliters of iodinated contrast material with a power injector at a rate of 3 milliliters per second through the antecubital vein. The anatomical range covered was from the diaphragmatic dome to the symphysis pubis. Images from the routine preoperative arterial and portal phases of the CT scan of each patient were reviewed by a radiologist that was blinded to patient- and operation-related characteristics and clinical outcome in terms of anastomotic complications. The presence, location, and grade of vascular calcifications in the arteries responsible for the anastomotic blood supply, (i.e., aorta, SMA, JVA, and LIPA) was assessed and reported. The grade of vascular calcification using a modified visual calcification grading system was based on a previously published study [9]. The arterial calcification grade system classifies abdominal aorta and SMA as three grade scales (zero = absent, 1 = minor, 2 = major) and the JVA and LIPA as two scales (zero = absent, 1 = present). Detailed definitions are reported in (Table 1), and examples of image characteristics are demonstrated in (Figure 1).

### 2.5. Statistical Analysis

The continuous variables, age, and BMI were converted into categorical variables. A Chi-square test was used to compare the variables between the two groups. *p*-Value < 0.050 was considered statistically significant. Data analysis was performed using IBM SPSS 24 software (SPSS Inc., Chicago, IL, USA).

## 3. Results

### Baseline Characteristics

The comparison of clinical, operative, and pathological variables after PSM showed no significant differences between the two groups. The overall mean age was 63.4 years and male patients comprised the majority in each group. The mean BMI was 23.1, with no underweight or obese patients in the complication group. A history of CVD or smoking was reported in 17 patients (73.3%) with EJA complications, and 16 (53.3%) without EJA complications, (*p* = 0.795). Five patients (16.7%) in the complication group and four (13.3%) in the non-complication group have a history of diabetes mellitus, (*p* = 0.718). Operative variables were similar between the groups, as each of them included 22 patients (73.3%) who underwent open gastrectomy, and five patients (16.7%) who needed completion gastrectomy after the diagnosis of remnant gastric cancer, (*p* = 1.000). The most common tumor location in the complication group was the upper third with 19 patients (63.3%) and the middle third in non-complication group with 14 patients (46.7%), (*p* = 0.149). The pathological stage I was predominant in both groups with 17 (57.7%), and 19 patients (63.3%) in the complication and non-complication groups, respectively, followed by stage III, (*p* = 0.365) (Table 2). 

Out of 60 patients, 28 (46.7%) had no calcifications, 24 (40.0%) had minor and eight (13.3%) had major calcifications in the aorta. SMA calcifications were absent in 40 patients (66.7%), minor in 15 (25.0%), and major in five (8.3%). No calcifications were detected in the JVA or LIPA in any of the 60 patients.

The comparison between the aortic and SMA calcifications and anastomotic complications was analyzed with two categories comprised of “absent/minor” and “major” calcifications.

As demonstrated in (Table 3), aortic calcifications were not associated with EJA complications. In the complication group, 25 patients (83.3%) had no, or minor calcifications and five patients (16.7%) had major calcifications. In the non-complication group, 27 patients (90.0%) had minor calcifications and three (10.0%) had major calcifications (*p* = 0.440). 

Major SMA calcifications were related significantly to anastomotic complications. Among 30 patients with anastomotic complications, five patients (16.7%) had major SMA calcifications, while in the non-complication group, no patients had major calcifications in the SMA (*p* = 0.020).

On analysis of the relationship between major SMA calcifications and the types of EJA complications (leak or stricture), three (13.0%) of 23 patients that were diagnosed with anastomotic leak had major SMA calcifications, and two (28.6%) of seven patients with stricture had major SMA calcifications (*p* = 0.028) (Table 4). 

## 4. Discussion

Anastomotic complications after total gastrectomy have a serious impact on the postoperative course of patients with gastric cancer. The anastomotic site requires sufficient blood supply for proper healing, and tissue ischemia is a common predisposing factor for both leak and stricture.

This study investigated the effect of vascular calcifications as a cause of tissue ischemia, on the development of EJA complications (leak, and stricture) after total gastrectomy. Furthermore, it examined the ability of preoperative CT scan calcification scoring to predict high-risk patients. The proposed visual calcification grading system is modified from a previously validated one, which was proven to be valuable in scoring aortic abnormalities and predicting cardiovascular events [9]. Unlike other calcium scoring techniques that require special software to determine the arterial calcium score and volume, this system is simple and can be easily interpreted by radiologists in daily clinical practice.

Identifying patients at risk of developing EJA complications with routine CT scan would be helpful in the perioperative management strategy. This could facilitate preoperative risk counselling and control particularly for the factors that might interfere with anastomotic blood flow. In addition, it might also prompt close postoperative monitoring and early follow up to perform appropriate timely interventions in these situations. This might add further benefit to the CT scan as a modality in the workup of gastric cancer in addition to TNM clinical staging and demonstration of perigastric vascular variations.

Our study could not evaluate the influence of JVA or LIPA calcifications on EJA healing, as none of the sixty patients had calcifications in these arteries. However, the association between aortic or SMA calcifications and EJA complications was determined. 

With regards to aortic calcifications, neither minor nor major grades were associated with EJA complications in this study. Previous studies of esophagogastric anastomosis are conflicting with some showing that aortic calcifications increase the risk of leak after esophagectomy [9,10,11]. and others show no such association [12]. The reason why aortic calcifications do not interfere with EJA anastomotic healing is not well understood. Nevertheless, this finding is consistent with the fact that the most common cause of chronic mesenteric ischemia (which might affect the jejunal anastomotic site) is atherosclerotic calcifications in the main branches of the abdominal aorta (i.e., celiac, superior mesenteric and inferior mesenteric arteries) rather than atherosclerosis in the aorta itself. [13]. This is in addition to its large diameter, which allows a high volume of intestinal blood flow even in the presence of calcifications. Moreover, the rich non-segmental submucosal intramural blood supply of the esophagus could prevent anastomotic ischemia on the esophageal side.

Of interest, our results showed a significant relation between EJA complications and major calcifications in the SMA, which is responsible for blood supply to the jejunal side of the anastomosis. Furthermore, major SMA calcifications were not only related to EJA leak, but were strongly related to stricture as well, indicating that tissue ischemia resulting from such calcifications might compromise anastomotic site healing.. These findings suggest that detecting major SMA calcifications by CT scan, which is considered a marker of systemic macrovascular atherosclerotic disease [14], is more indicative of anastomotic stricture than leak, and the role of generalized macrovascular disease in the pathogenesis of stricture plays a bigger role than localized microvascular impairment. A previous study found higher stricture rates after removal of larger-size vessels from the mesenteric arcade, likely owing to macrovascular impairment. This is in contrast to tight ischemic suture lines that resulted in microvascular impairment [15].

Although all reported major SMA calcifications were only in patients in the complication group, we cannot conclude that finding these calcifications on a CT scan is a definite indicator of EJA stricture. It should be taken into consideration that collateral mesenteric circulation between the major branches of the abdominal aorta plays a significant role in preventing bowel ischemia at the jejunal side of the anastomosis. It has been previously shown that this collateral network could develop more in the case of chronic stenosis or occlusion (e.g., in the celiac artery or SMA) which might be protective against EJA stricture in patients with major SMA calcifications [16].

On the other hand, tissue ischemia that leads to anastomotic leak was recognized to be a result of combined generalized vascular disease and inadequate local perfusion [9,10,11,12]. Impaired microcirculation due to cardiovascular conditions such as hypertension or coronary artery disease, or due to smoking, could lead to tissue ischemia and subsequent leak [6,17].

Local hypoperfusion is associated with anastomotic hypoxia and starts immediately after constructing the anastomosis. This was shown by a randomized prospective study to increase the incidence of EJA leak, especially if it persists in the first 24 h [18]. Severe EJA hypoxia is expected in the presence of major SMA calcifications and its negative impact on anastomotic healing could be aggravated by several possible mechanisms. First, the upward traction of the jejunal Roux limb to the diaphragmatic hiatus for gastrojejunostomy could change the main axis of the SMA, which typically travels in an anterior and inferior direction, and reduce blood flow to the anastomotic jejunal side due to compromised vascular conformability induced by atherosclerotic changes. Another mechanism is the possible damage to the mesenteric vascular supply of the jejunal bowel loop during its mobilization towards the esophagus using the surgical anastomosis stapler.

The importance of good anastomotic line perfusion in the prevention of leaks was demonstrated in several studies that showed promising results of lower rates of leak after esophagectomy by using intraoperative indocyanine green (ICG) evaluation for local microperfusion status before constructing the anastomosis [19,20]. Therefore, with regards to anastomotic leak, the presence of macrovascular calcifications on CT scan might not be enough to predict patients at risk, and one should consider using another method to assess the local anastomotic microperfusion, especially on the jejunal side, such as intraoperative ICG angiography with a near infrared (NIR) camera in order to prevent anastomotic leak in high risk patients, as this was found to be feasible in providing imaging to anastomotic blood flow in laparoscopic gastric cancer surgery [21].

The limitation of this study lies in the small sample size due to the relatively low incidence of EJA complications. However, it was possible to conclude that major SMA calcifications might induce tissue ischemia that compromises esophagojejunal anastomotic healing after total gastrectomy for gastric cancer and lead to anastomotic complications, particularly stricture, rather than leak.

## 5. Conclusions

In conclusion, vascular calcification could affect the condition of the esophagojejunostomy after total gastrectomy. Major SMA calcification is particularly associated with the stricture of EJA. A preoperative CT scan could predict patients at risk. Further studies are needed to evaluate advanced techniques, such as intraoperative ICG angiography, in preventing EJA complications in patients with major SMA calcifications.

## Figures and Tables

**Table 1:** Modified visual arterial calcification grading system.

Artery	Arteria Calcification Score		
	Score 0	Score 1	Score 2
**Aorta**	Absent	Minor calcifications: Nine or fewer foci and three or fewer foci extending over three or more sections	Major calcifications: More than nine foci or more than three foci extending over three or more sections
**SMA**	Absent	Minor calcifications: Extending over fewer than three sections or maximum cross-sectional diameter of single focus 10 mm or smaller	Major calcifications: Extending over three or more sections and maximum cross-sectional diameter of single focus, larger than 10 mm
**Jejunal**	Absent	One or more calcifications	Not applicable
**LIPA**	Absent	One or more calcifications	Not applicable

**Table 2:** Comparison of baseline characteristics between complication and non-complication groups after propensity score matching.

Variable	Complication Group (N = 30)	Non-Complication Group (N = 30)	*p*
Age group (years) <60 60–70 >70	7 (23.3) 16 (53.3) 7 (23.3)	12 (40.0) 9 (30.0) 9 (30.0)	0.172
Sex Male Female	23 (76.7)7 (23.3)	22 (73.3)8 (26.7)	0.766
BMI (kg/m^2^) Underweight (<18.5) Normal (18.5–22.99) Overweight (23.00–29.00) Obese (≥30)	0 (0.0) 15 (50.0) 15 (50.0) 0 (0.0)	1 (3.3) 14 (46.7) 14 (46.7) 1 (3.3)	0.558
CVD/Smoking Yes No	17 (56.7) 13 (43.3)	16 (53.3) 14 (46.7)	0.795
Approach Open Laparoscopic	22 (73.3) 8 (26.7)	22 (73.3) 8 (26.7)	1.000
Remnant stomach Yes No	5 (16.7) 25 (83.3)	5 (16.7) 25 (83.3)	1.000
Pathological stage I II III IV	17 (56.7) 5 (16.7) 7 (23.3) 1 (3.3)	19 (63.3) 1 (3.3) 8 (26.7) 2 (6.7)	0.365

BMI, body mass index; CVD, cardiovascular disease. Proportion ( ) are presented for categorical data.

**Table 3:** Association between arterial calcifications with esophagojejunal anastomosis complications.

Arterial Calcification Score	Anastomotic Complications (N = 30)	No Anastomotic Complications (N = 30)	*p*
Aorta			
0–1 (No/Minor) 2 (Major)	25 (83.3) 5 (16.7%)	27 (90.0) 3 (10.0)	0.440
SMA			
0–1 (No/Minor) 2 (Major)	25 (83.3) 5 (16.7)	30 (100) 0 (0.0)	0.020
Jejunal artery			
0 (Absent) 1 (Present)	30 (100) 0 (0.0)	30 (100) 0 (0.0)	N/A
LIPA			
0 (Absent) 1 (Present)	30 (100) 0 (0.0)	30 (100) 0 (0.0)	N/A

SMA, superior mesenteric artery; LIPA, left inferior phrenic artery. Proportions ( ) are presented for categorical data.

**Table 4:** The correlation between the kinds of anastomotic complication and the degree of SMA calcification.

**Anastomotic Complications**	**SMA Calcification Score (N = 60)**		***p***
	**0–1 (No/Minor)**	**2 (Major)**	**0.028**
Leak			
23 (38.3%)	20 (87.0%)	3 (13.0%)	
Stricture			
7 (11.7%)	5 (71.4%)	2 (28.6%)	
None			
30 (50.0%)	30 (100%)	0 (0.0%)	
Total			
60 (100%)	55 (91.7%)	5 (8.3%)	

SMA, superior mesenteric artery. Proportions ( ) are presented for categorical data.

## Data Availability

The data presented in this study are available on request from the corresponding author.
